# Editorial: Viral hypothesis in cardiac arrhythmias

**DOI:** 10.3389/fcvm.2022.1067501

**Published:** 2022-12-09

**Authors:** Michele Mario Ciulla

**Affiliations:** ^1^Smart Laboratory of Clinical Informatics and Cardiovascular Imaging, University of Milan, Milan, Italy; ^2^CLO, Department of Clinical Sciences and Community Health, University of Milan, Milan, Italy

**Keywords:** viruses, arrhythmias, SARS-CoV-2, cardiovascular disease, vaccination, myocarditis, pericarditis

All components of the heart, such as the pericardium, myocardium, endocardium, valves, autonomic nerves, and coronary arteries, might be unpredictably damaged during an infection caused by various microorganisms. Even if the mechanisms of the disease vary based on the species involved, it is generally accepted that damage is the result of an adverse interaction between a biological agent and a predisposed host caused directly by invasion/replication and cytokine/chemokine release, and indirectly by autoimmune mechanisms, including molecular mimicry or a reaction to the released/exposed host's antigens and/or other metabolic and genetic involvements (1).

When the inflammatory response is limited to the heart lining, it results in pericarditis, a generally benign process, even if recurring. When the cardiac muscle is involved, this causes myocarditis, which can be subclinical but also sometimes severe, causing acute heart failure and death (2). Patients with myocarditis and overlapping pericarditis, known as myopericarditis, usually report arrhythmias and ECG changes are often the only clinical manifestation (3).

The most common causes of pericarditis/myocarditis in developed countries are viruses, including the recent SARS-CoV-2 (3), and rarely bacteria, protozoa, fungi, parasites, toxic substances, drugs/vaccine-associated hypersensitivity and immune diseases such as sarcoidosis or systemic lupus erythematosus (4).

The incidence over time reflects the highly variable diffusion of viruses and the pre-existence of specific receptors in host tissues since the cellular entry is mediated by the recognition and binding of receptors (5). The spread and specificity of the receptors are therefore key factors for host diffusion, tissue tropism, pathogenesis, and the transmissibility of viruses (6).

Although more than 90% of the population is exposed to cardiotropic viruses, an adverse virus-host interaction seems to be a relatively rare phenomenon, since histologically-ascertained myocarditis is reported only in 1–5% of subjects (7). However, as there is no data from asymptomatics, this incidence might be underestimated.

As with influenza viruses, the COVID-19 pandemic, caused by the spread diffusion of SARS-CoV-2, represents a unique opportunity to explore possible links between viral infection and cardiovascular complications, including cardiac arrhythmias and, more recently, following reports of vaccine-related pericarditis/myocarditis, as cardiovascular side-effects of antiviral vaccination/treatments (8).

Cardiac arrhythmias could be defined as any ECG-documented variation from the normal heart rate and/or rhythm that is not justified under a physiological demand. According to the physiology of the heartbeat, two main mechanisms are supposed, i.e., enhanced/abnormal impulse formation and a reentry caused by conduction disturbances.

Indeed, in about 90% of COVID-19 critically ill patients, ECG abnormalities have been reported, among them, sinus tachycardia is the most frequent, followed by atrial fibrillation/flutter, ventricular arrhythmias, bradycardias, and ST-T changes (9). This high prevalence, excluding common causes like anxiety and pain, hypovolemia, hypoperfusion/hypoxia, and high body temperature, seen in any critical patient, is possibly related to the fact that SARS-CoV-2, by using angiotensin converting enzyme 2 receptor to enter cells by endocytosis (10) to become widespread in human tissues directly infects cardiomyocytes, causing cytokine activation, sarcomere disassembly, and myocardial cell death (11). Thus, cardiac arrhythmias in COVID-19 might be a multifactorial phenomenon where the pro-inflammatory cytokine cascade acts as a primer for apoptosis/necrosis/fibrosis and subsequent electrical remodeling (12).

The review by Sozzi et al. provides a contemporary overview of myocarditis, including classification, clinical implications, and treatment options supporting the role of cardiac magnetic resonance as the golden standard for diagnosis. Furthermore, the detection of necrosis/fibrosis at late gadolinium enhancement at baseline since in patients with acute myocarditis is associated with all-cause and cardiac mortality and major adverse cardiovascular events, including sustained ventricular arrhythmias, the extension and distribution of necrosis/fibrosis also have prognostic relevance.

In a prospective consecutive cohort of patients with COVID-19, the incidence of cardiac arrhythmias was 25%; patients with arrhythmias have a worse inflammatory status as demonstrated by higher CRP. Predictors of arrhythmia occurrence during a mean follow-up of 19 months were a more extensive pulmonary involvement and the need for oxygen therapy and CT severity score (Mouram et al.).

In a retrospective cohort study of hospitalized adult patients admitted with COVID-19 infection, Isakadze et al. support that significant elevations of the inflammatory marker CRP are independently associated with prolongation of the QTc interval in hospitalized COVID-19 patients. A markedly elevated CRP may therefore contribute to QTc prolongation, which may significantly increase arrhythmic risk.

By prospectively enrolling consecutive patients admitted for severe COVID-19, Lazzerini et al. demonstrate that systemic inflammatory activation can “*per se*” promote QTc prolongation *via* IL-6 elevation, leading to ventricular electric remodeling. Such modifications, despite being transitory, may significantly contribute to arrhythmic events and be associated with poor outcomes in COVID-19.

In conclusion, although it is not completely elucidated if viruses, due to their tropism and mechanisms of action, are direct and/or indirect causes of cardiac arrhythmias, when considering the overall exposure of humans to cardiotropic viruses, the pattern of infection/replication and host inflammatory response with the random onset of arrhythmias in a short time frame makes a possible interplay seem reasonable and, indeed, such an interplay has been confirmed in the recent COVID-19 pandemic by the reported high prevalence of cardiac arrhythmias.

Viruses may trigger cardiac arrhythmias by activating the pro-inflammatory cytokines and the related apoptosis/necrosis/fibrosis signaling and/or disrupting gene expression profiles involved in sarcomere function and causing heterogeneity of current conduction, shortening of action potentials, and induction of depolarization, especially in some predisposed phenotypes and/or where an underlying cardiovascular etiology already exists.

## Author contributions

The author confirms being the sole contributor of this work and has approved it for publication.
